# Fall injuries in Sub-Saharan Africa: analysis of prospective injury registry from 23 health facilities in Malawi and Tanzania

**DOI:** 10.1186/s12873-023-00805-x

**Published:** 2023-04-10

**Authors:** Hendry R. Sawe, Wakisa Mulwafu, Linda Chokotho, Kevin Croke, Rachel Chamanga, Meyhar Mohammed, Jonna Bertfelt, Harrieth P. Ndumwa, Juma A. Mfinanga, Sveta Milusheva

**Affiliations:** 1 Department of Emergency Medicine, Muhimbili University of Health and Allied Sciences, P.O. Box 65001, Dar es Salaam, +255 754 885 658 Tanzania; 2 Department of Emergency Medicine, Muhimbili National Hospital, Dar es Salaam, Tanzania; 3 Department of Surgery, Kamuzu University of Health Sciences, Blantyre, Malawi; 4 Malawi University of Science and Technology, Blantyre, Malawi; 5 Harvard T. H. Chan School of Public Health, Boston, MA USA; 6 Elizabeth Glaser Pediatric AIDS Foundation, Lilongwe, Malawi; 7 Development Impact Evaluation Department, World Bank, Washington, DC USA; 8 Muhimbili University of Health and Allied Sciences, Dar es Salaam, Tanzania

**Keywords:** Trauma burden, Epidemiology, Injuries in Malawi, Fall injuries, Injuries in Tanzania, Fall in Sub-Saharan Africa, Fall in LMIC

## Abstract

**Background:**

Low-and middle-income countries account for over 80% of fall-related fatalities globally. However there is little emphasis on the issue and limited high quality data to understand the burden, and to inform preventive and management strategies. We characterise the burden of fall injuries in Malawi and Tanzania.

**Methods:**

This multi-centre prospective descriptive study utilized trauma registry data from 10 hospitals in Malawi and 13 hospitals in Tanzania. The study included twelve months of data in Tanzania (October 2019 to September 2020), and eighteen months of data from Malawi (September 2018 to March 2020). We describe patient demographics, the causes, location, and nature of injuries, timing of arrival to hospital, and final disposition. Regression analyses were performed to determine risk factors for serious injuries.

**Results:**

There were 93,178 trauma patients in the registries of both countries, of which 44,609 (47.9%) had fall related complaints. Fall injuries accounted for 55.3% and 17.4% of all trauma cases in Malawi and Tanzania respectively. Overall the median age was 16 years (Interquartile range (IQR) 8–31 years), and 62.8% were male. Most fall injuries (69.9%) occurred at home, were unintentional (98.1%), and were due to a ground level fall (74.9%). Nearly half of patients (47.9%) arrived at a facility using public transport, with median arrival time of 10 h (IQR 8–13 h) from initial injury. Extremities (87.0%) were the most commonly injured region, followed by head and neck (4.4%). Overall 3275 (7.4%) patients had potentially serious injuries. Age > 60 years was associated with two times odds of having serious injuries than those < 5 years, and those sustaining injury at work (adjusted Odds Ratio (aOR) 1.95 95% CI; 1.56–2.43) or recreational areas (aOR 3.47 95% CI; 2.93–4.10) had higher odds of serious injuries compared to those injured at home.

**Conclusions:**

In these facilities in Sub-Saharan Africa, fall injuries accounted for a substantial fraction of all injuries. While most common in younger males, those aged 5–13 and over 60 years were more likely to have serious injuries. Most falls occurred at home, but serious injuries were more likely to occur at recreational and work areas. Future efforts should focus on preventive strategies to mitigate these injuries.

**Supplementary Information:**

The online version contains supplementary material available at 10.1186/s12873-023-00805-x.

## Background

Injuries are among the top ten leading causes of mortality in the world, and are projected to increase over the coming decades [1, 2]. The World Health Organization (WHO) estimates that injuries account for 4.4 million deaths globally, constituting nearly 8% of the deaths worldwide [3]. Falls are the second leading cause of unintentional injury deaths, after road traffic injuries, resulting in an estimated 684,000 deaths annually [4]. In addition, falls are responsible for approximately 38 million disability-adjusted life years lost per year; this is more years lived with disability than road traffic injuries, drowning, burns and poisoning combined [4]. Low-and-Middle Income Countries (LMIC) are disproportionately affected by fall injuries, accounting for over 80% of the fall-related deaths worldwide [3–5]. Fall injuries are of increasing concern worldwide; data from high-income countries shows a changing face of major trauma towards a more elderly population, with fall as a predominant mechanism of injury [6]. In most LMIC, the trend with age remains largely unknown, and given the relatively younger population in LMICs, the pattern is likely to be different.

In Sub-Saharan Africa (SSA) there is sparse data on the nature, incidence, risk factors, mechanisms, types, and outcomes of fall injuries. While most SSA countries are experiencing increasing incidence of traumatic injuries, most literature and interventions have focused on road traffic crashes [7–9]. In most SSA countries, the risk factors, mechanism of injury and types of injuries due to falls remain largely unknown, presenting a serious evidence gap and challenge in developing injury prevention strategies and policies as well as determining appropriate resources and training for health care providers.

Until recently, there have been few efforts to establish prospective trauma registries in most countries in SSA to capture injury data prospectively; most previous efforts have focused on routine hospital data or single hospital data that has been shown to be incomplete [10]. In both Tanzania and Malawi, trauma registries were recently introduced at 13 and 10 diverse health facilities, respectively, to capture data on a pilot Emergency Medical Services (EMS) system which was planned to be introduced in specific areas of each country [11, 12]. These registries have provided an opportunity to prospectively obtain detailed patient-level data on falls, as well as other types of trauma. A previous analysis showed that in the Malawi registry, falls were the single most common cause of trauma [13]. Therefore, in an effort to understand the burden of fall injuries in SSA, we combined the data from these two registries to produce a detailed description of the mechanisms, injuries, and characteristics of patients involved in falls to inform both management and prevention strategies.

## Methods

### Study setting

Malawi is a low-income country, with a population of 19.1 million people, life expectancy of 64 years, and Gross Domestic Product (GDP) per capita of $637 [14]. The country has a mixed public and private health system, with the public health care system being largely free at the point of service and organized in primary, secondary and tertiary levels [15]. Primary level health services are provided by health centres and community hospitals, while secondary level care is provided in district hospitals and Christian Health Association of Malawi facilities (a non-government not-for profit providing free health services) [16]. Tertiary level care is provided in central hospitals, which have specialist level health services at regional level. In light of the limited number of referral hospitals, district hospitals take care of a substantial amount of trauma patients, including serious and complicated cases.

Tanzania, which borders Malawi to the south, is a lower-middle-income country with a population of approximately 60 million people, life expectancy of 65 years, and GDP per capita of $1077. It also has a mixed health care system including both public and private providers. The public health care system is organized in a pyramidal model ranging from dispensaries at the bottom of the pyramid to national level hospitals at the top [15, 17]. Dispensaries, which serve as subdistrict (ward) level facilities, are expected to be operational during daytime only and lack operating theatre capabilities, while health centres operate 24 h with basic operating theatre capabilities (mostly focused on maternal services). District hospitals serve the district catchment, and operate 24 h with more resources than health centres (including x-rays and ultrasound), while Regional Hospitals provide referral level care with specialists and more resources than district hospitals. The national referral hospital is at the top of the pyramid and provides consultant level care with specialised orthopaedic services and diagnostics.

Both Malawi and Tanzania have an established referral system that links the lower-level facilities to secondary and tertiary hospitals, ensuring continuity of care for injured patients. However, there are significant limitations with respect to specialized trauma care, with only a handful of health facilities capable of providing specialised orthopaedic and neurosurgical care [18, 19]. There are no formal trauma care systems and also no formal pre-hospital care system for supporting care at the site of injury, with patients typically having to utilize informal means of transport to the nearest health facility in both countries.

In Malawi, the trauma registry (TR) was implemented from August 2018 to June 2021 at ten health facilities located along the main highway in the country, traversing across most of the north-south length of the country. These facilities included two referral hospitals, seven district hospitals, and one community hospital. In Tanzania, a TR was implemented from September 2019 to October 2020 in Emergency Units (EUs) of 13 public health facilities located within two kilometers of the highway; this involved four regional referral hospitals, three district hospitals, five health centres, and one dispensary [11, 12].

### Study design

This was a prospective observational study of all patients with fall injuries extracted from a dataset of all trauma patients who were captured in the previously described trauma registry (TR).

In both countries, the registries were set up as part of a collaborative initiative between the Governments of Tanzania, Malawi and the World Bank, with the main aim of understanding the health impacts of implementing a pilot emergency medical service (EMS) along busy highways with high burden of trauma from road traffic accidents (the A7 highway in Tanzania and M1 in Malawi) [11, 12]. In Malawi, all ten hospitals involved in this registry were located along the main north-south highway, known as the M1. The distance between the northern-and southern-most hospital was approximately 800 km. Five hospitals are located in the southern segment of the M1 between Lilongwe (the capital city) and Blantyre (the commercial hub). These five facilities were selected because they had been selected by the World Bank and the government to be part of the EMS pilot project. The other five hospitals are located in the northern segment, which is north of Lilongwe city [11]. These were chosen because they were comparable in size to the five facilities that were part of the EMS pilot and were similarly close to the M1 highway, but they were unlikely to be affected by the EMS pilot because they are located in a different region of the country. In Tanzania, the registry was set up at 13 health facilities that included 6 health facilities (2 Regional hospitals, 3 health centres and 1 dispensary) involved in the EMS implementation along the A7 highway (which connects the commercial city of Dar es salaam to the southern border of Tanzania), and 7 (2 Regional, 3 district hospitals and 2 health centres) additional facilities that were in a comparison group not part of the EMS implementation [12]. As part of the EMS pilot all the facilities that were on the intervention corridor did receive some minor renovation, equipping, staff training and ambulance allocation. The TR implementation, health facilities involved and data collection processes in Malawi and Tanzania are described in detail in separate publications [11–13]. Note that the data collection used for this study occurred prior to the implementation of the pilot EMS systems.

### Study population

All patients with fall as mechanism of injury were included in this analysis. The TR from all facilities collected data of all patients presenting with injury related complaints. The TR excluded patients who returned to the health facility for follow-up care or those appearing in the facility after referral from another study site, to avoid dual capture of the same patient.

### Study procedure and data source

At all sites, TR collected data on patient demographics, mode of transport to hospital, geographic location of trauma, injury details, injury severity, management, care outcomes, diagnosis and time attended, setting, intent and final disposition of the patients.

In Tanzania, a paper-based standardised trauma documentation form was used for both clinical care and to populate the TR [12]. Research assistants (RAs) were recruited for each health facility in Tanzania to support the data collection process. The RA transferred the data to online software Research Electronic Data Capture (REDCap) (© REDCap version 7.2.2, Vanderbilt, Nashville, TN, USA). In Malawi, TR data was collected using a digital trauma registry tool using Computer Assisted Personal Interview format via tablets, programmed by the research team using SurveyCTO (©Dobility, Inc, Washington DC, USA). Details on implementation of the registry and data collection procedure are presented in a separate manuscript [11].

### Study duration

In both countries, prior to initiating formal data collection, we conducted pilot studies in August 2018 (in Malawi) and September 2019 (in Tanzania); these data are excluded in the current analysis. We have also excluded data from April 2020 in the Malawi TR when COVID-19 impacted the health care facility access, and data from October 2020 in Tanzania TR due to partial capture as the data collection activities were ending. In this analysis, we present Malawi TR data from September 2018 to March 2020 and Tanzania TR data from October 2019 to September 2020.

### Data analysis

Data from both countries were exported from online platforms (REDCap and Survey CTO) and imported into STATA version 17, StataCorp, College Station, Texas, USA, then cleaned, coded and analysed. Descriptive statistics are reported on demographics, mechanisms of fall, location of injury, severity of injury, referral pattern and final disposition using frequencies, percentages, median and IQR. Logistic regression analyses were performed and the odds ratio with the corresponding 95% Confidence Interval (CI) were used to determine associations between nature of fall injuries with risk factors for serious injuries. We defined serious injuries as a need for hospitalization (including intensive care unit (ICU) and operating theatre (OT)) and transfer to higher-level facilities or death. **Variables with significant unadjusted odds ratio (UOR) (using a cut off** ***p-value of < 0.05) were included in the multivariate regression analysis model. A p-value of < 0.05 was considered to be statistically significant***. **In order to understand the seasonal and regional variability of fall injuries, we plotted a proportion of fall injuries per each month and district in which the injury has** happened.

## Results

### Baseline characteristics of patients

There were 93,178 trauma patients in the registries of both countries. During the reporting period, the TR in Tanzania captured a total of 18,175 trauma patients in all facilities, out of which 3,169 (17.4%) had fall-related injuries. In Malawi, the TR recorded 75,003 injured patients of which just above half 41,440 (55.3%) had fall-related injuries. Overall, a total of 44,609 (47.9%) patients with fall injuries in both countries were included in this analysis. For the sample as a whole, 28,005 (62.8%) were male, and the overall median age was 16 years with interquartile range of 8 to 31 years. Patients within age groups 5–13 years (30.7%), and 14–24 years (23.8%) had the highest percentage of fall injuries, while those ≥ 60 years had (4.6%). More than a third (43.7%) of fall patients were engaging in agriculture or manual labor activities. In both countries, there was variability in injury incidents per month without a clear pattern; furthermore, five districts in each country accounted for over half of the fall injuries recorded (Figure Sup1 and 2). The geographic dispersion of cases is largely driven by the location of the health facilities, which hosted the registries.

There were a number of differences in the social and demographic characteristics of patients seen in these two countries. In Tanzania, there were more patients over 60 years of age than in Malawi (13.5% vs. 3.9% respectively). Trauma patients in Malawi were more likely to report agriculture and manual labor as their job (45.4%) compared to Tanzania (29.6%), although a larger percentage of patients in Malawi had attained higher education (29.8%) compared to Tanzania (19.3%). With regard to transport, in Tanzania a majority of patients arrived by a motorized two or three-wheeler (53.5%) whereas commercial cars were the most common methods of transport in Malawi (50.3%). Furthermore, in Tanzania (0.3%) came by bicycle compare to (12.2%) in Malawi. Most patients in Tanzania (67.8%) were seen at Regional level hospitals, while in Malawi most (69.8%) were seen at District level hospitals (Table 1).

**Table 1:** Patient characteristics

	Overall (N = 44,609)	Malawi (n = 41,440)	Tanzania (n = 3169)
**Gender**	n (%)	%	%
Male	28,005 (62.8)	62.7	64.3
Female	16,604 (37.2)	37.3	35.7
**Age groups**			
< 5 years	5287 (11.9)	11.7	14.6
5–13 years	13,690 (30.7)	30.9	27.7
14–24 years	10,621 (23.8)	24.5	15.2
25–40 years	8248 (18.5)	18.6	16.5
41–50 years	2853 (6.4)	6.3	7.3
51–60 years	1864 (4.2)	4.1	5.2
≥ 60 years	2046 (4.6)	3.9	13.5
**Age**			
Median (IQR) years	16 (8–31)	16 (8–30)	18 (7–41)
**Level of Education** ^b^	N = 35,577	n = 33,194	n = 2383
No formal education	2026 (5.7)	5.3	11.8
Primary School	22,097 (62.1)	61.9	65.1
Secondary School	10,341 (29.1)	29.8	19.3
Vocational or college or University	830 (2.3)	2.3	2.6
Unknown	283 (0.8)	0.8	0.8
**Occupation** ^♮^	N = 22,759	n = 21,126	n = 1633
Agriculture or Manual labor^1^	10,311 (45.3)	45.4	49.6
Technical or Professional work^2^	3314 (14.6)	13.8	26.3
Student	4697 (20.6)	21.5	9.6
Unemployed^3^	4157 (18.3)	18.7	12.1
Other^4^	280 (1.2)	0.5	1.65
**Transport to health facility**	N = 44,575	n = 41,432	n = 3143
Motorized (two or three) wheeler	5971 (13.4)	10.4	53.5
Bicycle	5014 (11.3)	12.1	0.3
Ambulance	1099 (2.5)	2.5	2.4
Walk-in	7320 (16.4)	16.9	10.5
Private car	3390 (7.6)	6.9	16.1
Commercial car^5^	21,328 (47.9)	50.3	16.2
Others^6^	453 (1.0)	1.0	1.08
**Level of facility** ^*7*^	N = 44,609	n = 41,440	n = 3169
Regional/Central	14,676 (32.9)	30.2	67.8
District	28,556 (64.0)	68.3	8.9
Health Centre^*8*^	1315 (2.9)	1.5	21.3
Dispensary	62 (0.1)	0	1.9

^*1*^ *Includes; Agriculture, labor, Craftsman, Domestic worker, Driver, Mining*

^*2*^ *Includes;Health care worker, Military, Office worker, Petty trade, Police, Army, Security and Teacher*

^*3*^ *Includes;Housewife, Retired and Unemployed*

^*4*^ *Includes; Pastor, Priest, Prisoner*

^*5*^ *Includes; Bus, Commercial Vehicle, Mini Bus, Public Transport*

^*6*^ *Includes; Lorry, Oxcart, Police, Truck*

^*7*^ *Function of what facilities were included in the analysis in each country*

^*8*^ *In Malawi this was a Community Hospital*

^b^ *Excludes patients aged < 7 years |* ^δ^ *Excludes patients aged < 16 years*

### Description of nature of injuries

Overall, three quarters 29,100 (74.9%) of all patients sustained ground level falls (while walking, running or playing), with home being an injury location for over two thirds (69.9%) of all fall patients. Few patients (less than 2%) self-reported to be under the influence of alcohol at the time of sustaining fall injury. Median time of arrival at the health facility was 10 h after the fall, with more patients from Tanzania arriving ≥ 12 h after sustaining fall injury. It is important to note that this time represents both travel time to the facility as well as the time patients wait before deciding to seek medical care. 3,275 patients (7.4%) had potentially serious injuries requiring hospitalization, transfer to higher-level facilities, or died while receiving care in EU. There was a large difference in proportion of serious cases in Tanzania (47.7%) versus Malawi (4.3%), which reflects the greater focus of the Tanzania registry on emergency care versus the Malawi registry’s focus on all trauma cases. (Table 2)

**Table 2:** Description of nature of injuries

	Overall	Malawi	Tanzania
	N = 38,849	n = 37,804	n = 1045^*ф*^
**Mechanism of fall**	n (%)	%	%
Fall from height	9456 (24.3)	23.6	51.8
Ground level fall*	29,100(74.9)	75.7	48.0
Others^*1*^	293 (0.8)	0.8	0.2
**Activity/Location of fall**	N = 44,468	n = 41,320	n = 3168
Work	2168 (4.9)	4.5	9.9
Home	31,077(69.9)	70.3	64.6
Street/public place	7803 (17.6)	17.4	19.9
Recreational	3420 (7.7)	7.9	5.1
**Injury intent**	N = 44,155	n = 41,419	n = 2736
Unintentional	43,328 (98.1)	98.0	99.7
Intentional	827 (1.9)	1.9	0.3
**Alcohol status/use** ^*2*^	N = 42,331	n = 41,438	n = 893
No	41,606 (98.3)	98.3	96.3
Yes	541 (1.3)	1.2	3.7
Suspected	184 (0.4)	0.4	0
**GCS at presentation** ^*ρ*^	N = 36,281	n = 33,804	n = 2477
Normal GCS	31,411 (86.6)	87.3	77.2
Mild TBI**	1523 (4.2)	3.1	19.3
Moderate TBI	1657 (4.6)	4.7	3.1
Severe TBI	1690 (4.7)	5.0	0.4
**Referral status**	N = 44,563	n = 41,440	n = 3123
Direct from injury site	36,888 (82.8)	82.5	86.5
Referred from another facility	7675 (17.2)	17.5	13.5
**Hospital arrival time**			
Median (IQR) hours	10 (8–13)	10 (8–13)	12 (10–16)
**Hospital arrival time**	N = 44,609	n = 41,440	n = 3166
< 6 h	711 (1.6)	1.4	1.6
6–11 h	28,186 (63.2)	65.0	39.4
12-17 h	12,557 (28.2)	27.3	39.2
≥18 h	3,152 (7.1)	6.3	17.7
**Disposition**	N = 44,525	n = 41,380	n = 3,145
Discharged Home	41,250(92.64)	95.7	52.3
Admitted (*include ICU and OT*)	2,886 (6.5)	4.2	37.1
Referred	376 (0.84)	0.1	10.4
Died at EU	13 (0.03)	0.02	0.2

*Includes fall while walking, running or playing

**Traumatic brain Injury

^*1*^*Includes; cliff, deep hollow, hill, electric pole, pit, graveyard, ditch*.

^*2*^ *Self reported or suspected.*

^*ρ*^*Average for adults only*.

^*ф*^*Only added at a later stage of TR implementation hence earlier patients were not asked this question*.

### Characteristics of injuries by age group

Males constituted the majority of fall injuries across all age groups except for those with more than 60 years (41.6%). Among those who had ground level fall (n = 29,100), age group 5–13 years and ≥ 60 years accounted for the highest 8921 (30.7%) and lowest 1342 (4.6%) proportion respectively. Extremities were the most common region of injury 87.0% and were mostly sustained by a third (31.3%) of children with 5–13 years. Among those that sustained chest injuries, 46.1% of them were aged 25–59. The majority (92.6%) of patients was discharged home. However, one-fifth (19.5%) of elderly patients (≥ 60 years) were admitted **(**Table 3**).**

**Table 3:** Characteristics of fall injuries by age group

	Overall	< 5 years	5–13 years	14–24 years	25–59 years	≥ 60 years
	N = 44,609	n = 5287	n = 13,690	n = 10,621	n = 12,680	n = 2331
**Gender**	n (%)	%	%	%	%	%
Male	28,005 (62.8)	57.4	66.3	71.8	57.5	41.6
Female	16,604 (37.2)	42.6	33.7	28.2	42.5	58.4
**Mechanism of fall**	N = 38,849	n = 4505	n = 11,785	n = 9425	n = 11,286	n = 1848
Fall from height	9456 (24.3)	19.5	23.9	19.7	30.3	26.2
Ground level fall	29,100 (74.9)	79.8	75.7	79.6	68.6	72.7
Others	293 (0.8)	0.7	0.3	0.8	1.2	1.1
**Location of fall**	N = 44,390	n = 5200	n = 13,654	n = 10,595	n = 12,616	n = 2325
Work	2090 (4.7)	0	1.3	3.9	11.0	4.9
Home	31,077 (70.0)	92.1	71.3	55.4	69.3	83.3
Street or public place	7803 (17.6)	7.2	22.3	20.9	15.0	11.5
Recreational	3420 (7.7)	0.7	5.1	19.8	​​4.6	0.3
Region of injury*	N = 42,508	n = 4961	n = 13,114	n = 10,285	n = 12,075	n = 2073
Head and Neck	1949 (4.6)	5.4	4.3	4.1	5.2	3.4
Chest	653 (1.5)	0.5	0.9	1.3	2.5	3.4
Abd. Pelvic injury	357 (0.8)	0.3	0.5	0.8	1.2	1.9
Extremities	38,827 (91.3)	90.9	92.5	92.0	89.8	90.7
Others	1276 (3.0)	3.4	2.7	3.2	3.1	2.7
Unknown	65 (0.2)	0.1	0.1	0.1	0.2	0.05
**Disposition**	N = 44,525	n = 5280	n = 13,667	n = 10,600	n = 12,656	n = 2322
Discharged Home	41,250 (92.6)	93.7	91.4	94.9	94.1	78.9
Admitted**	2886 (6.5)	5.3	7.7	4.6	4.9	19.5
Referred	376 (0.8)	1.0	0.9	0.5	​​0.9	1.6
Died at EU	13 (0.03)	0.02	0.0	0.01	0.05	0.04

* *One patient may have sustained injury in one or more regions*

** *includes admission to general wards, Intensive Care Unit and Operating Theatre*

### Risk factors for serious injuries

Overall, patients between 5 and 13 years had 20% higher odds of having serious whereas those aged above 60 years had two times the odds of injury compared to those who were below the age of 5 years. (Table 4) Furthermore, those who had injuries at work had 2 times higher odds of serious injuries compared to those who sustained injuries at home. Patients who self-reported to be under the influence of alcohol during the fall had 60% higher odds (aOR: 1.60, 95% CI; 1.11–2.30, p = 0.012) for serious injuries compared to those who did not report to have drunk alcohol. Late hospital arrival time (≥ 18 h post fall) was associated with about 2 times higher odds for serious injuries. We also conducted country-specific sub-analysis of the data: in Malawi, the multivariate analysis showed that the age group, fall mechanism, fall location, alcohol status, hospital arrival time, and regions of injury were statistically significant risk factors for serious injuries. However in Tanzania only head and neck, and extremities injuries were statistically significant predictors of serious injuries (Table 4).

**Table 4:** Risk factors for serious injuries

	Overall					Malawi	Tanzania
Variable	N = 44,525	Non-discharged	Unadjusted OR UOR (95%CI)	Adjusted OR AOR (95%CI)	P value	Adjusted OR AOR (95%CI)	Adjusted OR AOR (95%CI)
Gender							
Male	27,950	7.7	1.16 (1.07–1.24)	1.07 (0.96–1.19)	0.236	1.05(0.94–1.19)	0.91(0.70–1.38)
Female	16,575	6.8	Ref	Ref		Ref	Ref
**Age groups**	**N = 44,525**						
< 5 years	5280	6.3	Ref	Ref		Ref	Ref
5–13 years	13,667	8.6	1.40 (1.23–1.58)	1.21 (1.01–1.44)	0.037	1.42(1.16–1.74)	0.91(0.57–1.46)
14–24 year	10,600	5.1	0.60 (0.69–0.92)	0.48 (0.39–0.59)	< 0.001	0.54(0.43–0.68)	0.71(0.40–1.28)
25–59 years	12,656	3.3	0.93 (0.82–1.07)	0.49(0.40–0.60)	< 0.001	0.52(0.41–0.65)	0.68(0.40–1.13)
≥ 60 years	2322	19.3	3.98 (3.42–4.62)	1.96 (1.56–2.45)	< 0.001	2.13(1.64–2.76)	1.16(0.58–2.28)
**Mechanism of fall**	**N = 38,802**						
Fall from height	9443	7.8	1.81 (1.65–1.99)	1.51 (1.35–1.69)	< 0.001	1.34(1.18–1.52)	0.70(0.50-1.00)
Ground level fall	29,067	4.4	Ref	Ref		Ref	Ref
Others^*1*^	292	5.8	1.33 (0.04–0.05)	1.04 (0.62–1.79)	0.872	1.00(0.56–1.78)	N/A**
**Location of fall**	**N = 44,384**						
Work	2161	12.3	2.03 (1.77–2.32)	1.95 (1.56–2.43)	< 0.001	2.28(1.79–2.90)	0.74(0.39–1.38)
Street or public place	7779	8.2	1.29 (1.18–1.42)	1.80 (1.59–2.04)	< 0.001	1.85(1.61–2.13)	0.96(0.64–1.45)
Recreational area	3410	10.1	1.621(1.44–1.83)	3.47 (2.93–4.10)	< 0.001	4.07(3.37–4.81)	0.77(0.37–1.58)
Home	31,034	6.5	Ref	Ref		Ref	Ref
**Alcohol status**	**N = 42,331**						
Confirmed	541	11.9	2.60 (2.0-3.39)	1.60 (1.11–2.30)	0.012	2.04(1.38–3.01)	0.73(0.18–2.97)
Suspected	184	8.7	1.85 (1.10–3.09)	1.52 (0.81–2.88)	0.191	1.70(0.89–3.37)	N/A**
No	41,606	4.9	Ref	Ref		Ref	Ref
**EU arrival time**	**N = 44,523**						
< 6 h	710	17.8	Ref	Ref		Ref	Ref
6–11 h	28,150	4.5	0.22 (0.18–0.27)	0.35 (0.25–0.49)	< 0.001	0.40(0.28–0.58)	0.77(0.28–2.15)
12-17 h	12,531	9.8	0.50 (0.41–0.61)	0.68 (0.49–0.95)	0.026	0.77(0.53–1.13)	0.65(0.23–1.81)
≥18 h	3132	20.6	1.21(0.18–0.26)	1.70 (1.21–2.40)	0.002	2.16(1.47–3.17)	0.81(0.28–2.32)
**Region of Injury**	**44,525**						
Head and Neck injury	1945	9.6	1.35 (1.16–1.58)	0.73 (0.57–0.93)	0.012	2.59(1.90–3.53)	7.60(2.34–24.56)
No Head & Neck injury	42,580	7.3	Ref	Ref		Ref	Ref
Chest injury	651	12.3	1.81(1.43–2.29)	1.15 (0.82–1.60)	0.83	4.37(2.98–6.43)	N/A**
No Chest injury	43,874	7.3	Ref	Ref		Ref	Ref
^*1*^Pelvic injury	357	21.3	3.46 (2.68–4.47)	1.97 (1.36–2.86)	< 0.001	6.84(4.43–10.56)	N/A**
No Pelvic injury	44,168	7.2	Ref	Ref		Ref	Ref
Extremities injury	38,786	5.9	0.30 (0.28–0.33)	0.49 (0.41–0.58)	< 0.001	2.40(1.78–3.23)	4.50(3.10–6.52)
No Extremities injury	5,739	17.2	Ref	Ref		Ref	Ref

**Dropped by the model as a default due to multicollinearity resulting from small sample size

^*1*^*Includes abdominal and pelvic injuries*.

### Characteristics of work-related fall injuries

Overall, 1776 (4%) of patients reported to have work-related fall injuries. These injuries were more common among male patients 1381(77.8%). The largest age category in this group of patients 829 (46.7%) was 25–40 years. Agriculture or Manual labor workers constituted more than two thirds 1,134 (66.2%) of work-related injuries. There were large differences between countries with regard to disposition, with 94.5% of patients with work injuries discharged home in Malawi and 52% of patients with work injuries discharged in Tanzania, including 12% who were referred to a higher level of care (Table 5).

**Table 5:** Characteristics of work related fall injuries

	Overall	Malawi	Tanzania
Gender	N = 1776	n = 1504	n = 272
Male	1381(77.8)	78.0	76.5
Female	395 (22.2)	22.0	23.5
**Age groups**	N = 1,776	n = 1504	n = 272
14–24 year	389(21.9)	22.5	18.4
25–40 years	829(46.7)	46.0	50.4
41–50 years	328(18.5)	18.4	18.8
51–59 years	147(8.3)	8.6	6.6
≥ 60 years	83(4.7)	4.5	5.9
**Mechanism of fall**	N = 1,425	n = 1313	n = 116
Fall from height	486 (34.1)	30.2	76.7
Ground level fall	896(62.9)	66.5	23.3
Others^*1*^	43(3.0)	3.3	0
**Occupation**	N = 1,700	n = 1443	n = 257
Agriculture or Manual labor^1^	1,161 (68.3)	70.7	54.7
Technical or Professional work^2^	439 (25.8)	23.3	40.1
Student	82 (4.8)	5.1	3.2
Other^3^	18(1.1)	0.9	1.9
**Region of injury**	N = 1776	n = 1504	n = 272
Head and Neck	141 (7.9)	8.8	3.3
Chest	81 (4.6)	5.2	1.1
Abdominal pelvic	48 (2.7)	2.7	2.6
Extremities	1428 (80.4)	89.6	29.4
Others	82 (4.6)	5.5	0
Unknown	8 (0.5)	0.3	0
**GCS at presentation**	N = 1720	n=​​1498	n = 222
Normal GCS	1520 (88.4)	90.7	73
Mild TBI	131 (7.6)	5.1	24.8
Moderate TBI	31(1.8)	1.8	1.8
Severe TBI	38 (2.2)	2.5	0.5
**Disposition**	N = 1,770	n=​​ 1499	n = 271
Discharged Home	1,558(88.0)	94.5	52.0
Admitted to hospital^*4*^	167(9.4)	4.7	35.4
Referred	42(2.4)	0.6	12.2
Died at EU	3(0.2)	0.1	0.4

^*1*^ *Includes; Agriculture, labor, Craftsman, Domestic worker, Driver, Mining*

^*2*^ *Includes;Health care worker, Military, Office worker, Petty trade, Police, Army, Security and Teacher*

^*3*^ *Includes; E-Work, sex worker, thief, cyclist and students*

^*4*^ *Includes those admitted to general wards, ICU and operating theatre*

## Discussion

This paper reports findings from prospective trauma registries set up in multi-level health facilities to support the development of EMS services in specific locations within Malawi and Tanzania [11, 12]. In this multicenter study we found a substantial burden of fall injuries, accounting for the first and second most common mechanism of injury in Malawi and Tanzania respectively. Similar to previous studies, males are more affected than females, constituting over 60% of all injuries. Part of the reason might be the male preponderance on certain social-economic activities, as well as differing nature of recreational activities between males and females in this context [20, 21]. In both countries, fall injuries are concentrated in children and young adults, with two-third of injuries occurring in those below the age of 24 years.

Prior studies of paediatric injuries have reported lack of oversight, and inability of children to appreciate and avoid predisposing injury situations as some of the common reasons and risk factors for injuries in this population [22, 23]. In our study the peak age of injury is between 5 and 24 years, with the majority of injuries happening in a home setting, a finding similar to prior studies in similar settings [22]. Our finding on the high incidence of fall injuries among young people differs from observed patterns in high-income countries, where recent emphasis has been on falls most commonly affecting the elderly population [6]. These findings underscore the importance of developing locally driven injury prevention interventions, such as those that focus on domestic and recreational settings, as well as increasing the knowledge of caretakers regarding prevention of fall injuries among children.

Ground level fall was the most frequent mechanism among all patients, while fall from height accounted for one-quarter of all the fall injuries. Furthermore, the majority of cases in Tanzania (over 50%) were due to fall from height while in Malawi, the majority of cases (over 75%) were due to ground level fall, we believe this might have been contributed by the fact that in Malawi, the terrain is not even or smooth and this might increases the risk of falls especially in children who are unlikely to pay attention when they are walking or playing. Furthermore, while our study did not record information about the impact of height of fall, fall from height is associated with more serious injuries and long-term impact [24]. Therefore, the much higher percentage of fall from height cases in Tanzania might partly explain the high proportion of patients having potentially serious injuries in Tanzania compared to Malawi. Nearly all injuries were unintentional, and use of alcohol prior to an injury was reported in less than 2% of the population, contrary to findings seen in other mechanisms of injuries [25].

Both countries lack formal pre-hospital services [26], which limits the ability of the health care systems to provide emergency care across its three fundamental domains of scene, transport and facility. We found that only 2% of patients were brought to hospital by formal ambulance services, while the majority utilized private vehicles to travel to health care facilities following an incident of injury. Interestingly, over 10% of patients in Malawi came by bicycle, compared to less than 1% who used bicycle in Tanzania. The absence of a formal pre-hospital service may predispose patients to both delays in making a decision to seek healthcare and reaching care, as well as increasing the likelihood of aggravated injuries or death due to patient mishandling by the lay providers [27]. Over 80% of patients arrived to health facilities directly from the injury site, and overall one-third of patients arrived at or after 12 h following fall injuries. While lack of pre-hospital processes may contribute to this timing of arrival at the facility, patient lack of awareness about the need to seek immediate medical care after injuries, and instead waiting until injuries are much worse, might have contributed to patients with severe injuries presenting late at the health facilities. Consequently, we found that those patients who arrived after eighteen hours had a two-fold likelihood of serious injuries compared to those who arrived within six hours of injuries.

Injuries to the extremities – including fracture of long bones, laceration and dislocations –were the most common across all age groups, while injuries to the head and neck were the second most common, constituting less than 5%, in concordance with prior studies of injuries in similar settings [12]. Injuries to the extremities, in particular fractures of the long bones, require specialised care to ensure short time to recovery as well as resumption of full functionality of the affected limb. In both countries, the specialised orthopaedic services are limited to tertiary referral facilities, at the peak of the health care pyramid, which render it challenging for most patients to access those services in a timely manner [18, 28]. Further evaluation of the severity of these injuries, and path to specialised care will be important so as to allow for proper planning of infrastructure and human resources appropriate for the level of care in each facility.

While a majority of patients were discharged home from health facilities, 7% of patients seen had serious injuries requiring hospitalization or transfer to higher-level facilities, or else died while receiving care in the facility. We found that paediatric (aged 5 to 13 years) and elderly patients above 60 years had 20% higher odds of potentially serious injuries, a finding that is similar to previously published studies of injuries in different age groups [29]. Similarly, while exposure to alcohol prior to injury was rare, those who had been under the influence of alcohol during the fall had 60% higher odds of serious injuries. Our sub-analysis of individual countries showed that the age group, fall mechanism, fall location, alcohol status, hospital arrival time, and regions of injury were statistically significant risk factors for serious injuries in Malawi. However, in Tanzania, only head and neck, and extremities injuries were statistically significant for serious injuries; this could reflect the small sample size of Tanzania compared to that of Malawi.

In our evaluation of a subset of patients who reported to have work related injuries, we found similar patterns in sex, region of injury, disposition and age as to the general population. A majority of work related injuries resulted from agriculture activities and manual labor, which is not surprising given the economic activities of the population in both countries. We propose further evaluation of the nature of the injuries sustained in these settings so as to inform appropriate interventions that will cover a broad population given that most people engage in and depend on economic activities related to agriculture.

Most prior studies of trauma in the region have reported widely about the general burden of injuries in single sites or sites of similar level of care, or else have focused on the impact of injuries resulting from road traffic crashes [12, 28, 30, 31]. Given the lack of fall injury specific data and evidence of resulting outcomes in low-income countries, the trauma registries used in these two countries provide a notable example of how important such registries are in informing the evolution of emergency care in low- and lower middle-income countries like Malawi and Tanzania. Moreover, data collected in these registries will be useful to provide baseline information and monitor improvements following interventions done to change the profile and reduce the burden of fall injuries in the two countries. The registries can be further updated to include data that will be useful to assess more outcome measures such as height of fall in different populations and associated impact.

## Limitations

The findings of our study are limited by the lack of detailed information on the final hospital outcome, and health outcomes of the patients after discharge from the hospital. Also, we are unable to report on the exact height of fall from patients who reported to have fallen from height and hence unable to comment on impact of various height related injuries. There was a small likelihood of social desirability bias by clinicians when RA inquired for additional trauma information and management, however presence of RA in Emergency Unit (EU) in real time reduced this likelihood. Our analysis included eighteen-months of data from Malawi and only one-year of data from Tanzania, which limited comparative sub-analysis for some variables from the Tanzanian data. In addition, observations for some variables were missing. In general, few observations were missing for most variables with the exception of the mechanism of the fall for Tanzania because this variable was added halfway through the data collection. We do not expect this to lead to bias though because after the variable was added it was consistently asked of all patients. Lastly, the data collection during registry implementation was impacted by the COVID-19 pandemic in various ways in both countries, including the need to withdraw the research assistants and have them working from home to minimize the risk of disease transmission during the later periods of data collection.

## Conclusion

In these facilities in Sub-Saharan Africa, fall injuries accounted for substantial burden among all injuries. While most common in younger aged males, those 5–13 and over 60 years were more likely to have serious injuries. Most falls occurred at home, but serious injuries were more likely to occur at recreational and work areas. Future studies should focus on testing various preventive strategies to mitigate the impact of fall injuries in these settings.

## Electronic supplementary material

Below is the link to the electronic supplementary material.

Supplementary Material 1

## Data Availability

The datasets used and/or analysed during the current study available from the corresponding author on reasonable request.
