# Supplementary material for: Fall injuries in Sub-Saharan Africa: analysis of prospective injury registry from 23 health facilities in Malawi and Tanzania
Source: BMC Emerg Med. 2023 Apr 10;23:42. doi: 10.1186/s12873-023-00805-x (PMC10088193; doi:10.1186/s12873-023-00805-x)

**Figure S1a:** Fall injuries distributed by district in Malawi


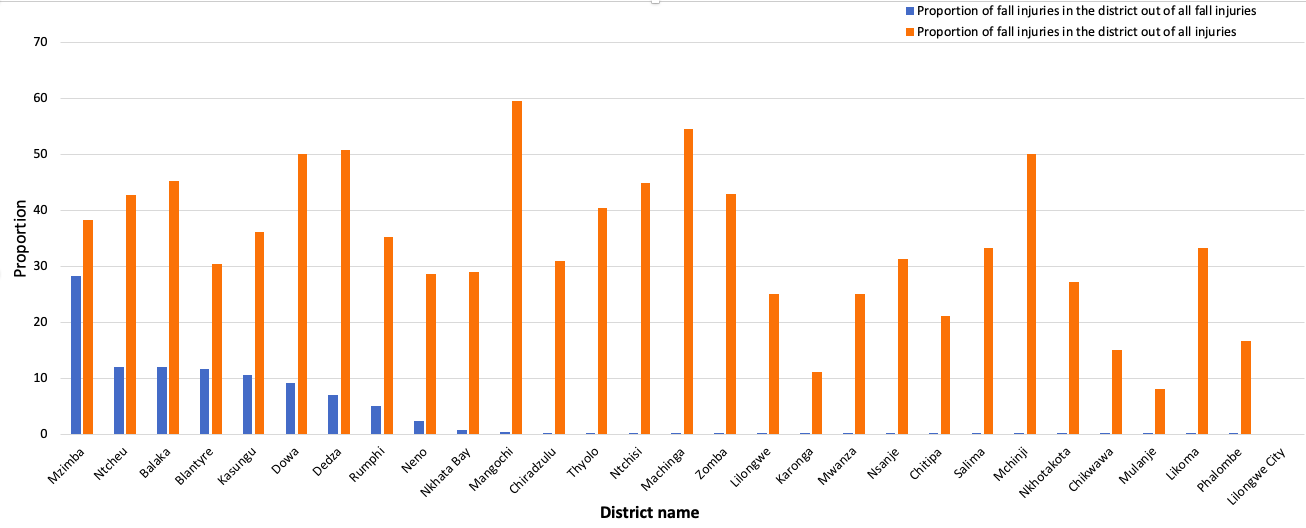


*-Overall, 42% of injuries had documented district name for injury settings.*

*-Overall, less than 20 injuries were recorded in, Mchinji, Salima, Nkhotakota, Nsanje, Likoma, Chitipa, Phalombe and Lilongwe districts.*

**Figure S1b:** Fall injuries distributed by district in Tanzania


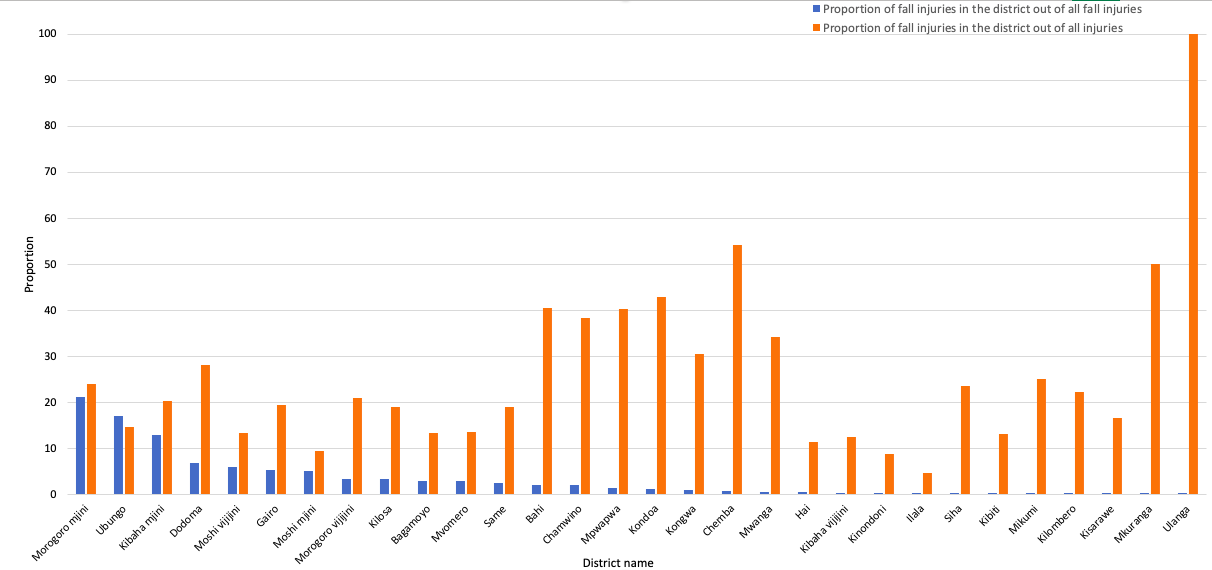


*-Overall, 74% of injuries had documented district name for injury settings.*

*-Overall, less than 20 injuries were recorded in Kigamboni, Kilombero, Kisarawe, Mikumi, Mkuranga, Rufiji, Siha, and Ulanga districts. Also, Rombo (n=22) and Temeke (n=53) districts had no records of fall related injuries.*

**Figure s2a:** Number of injuries distributed by months in Malawi


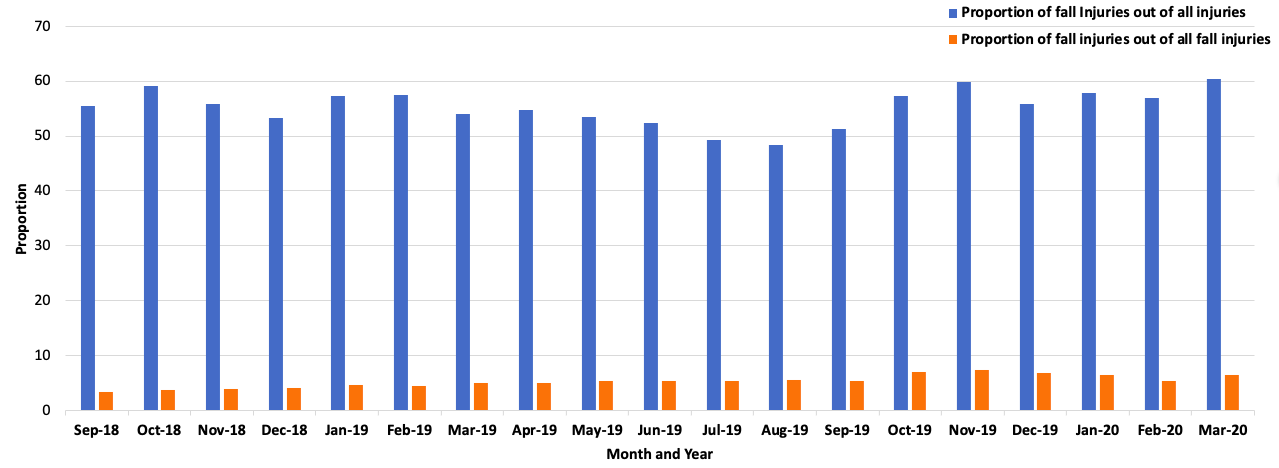


**Figure s2b:** Number of injuries distributed by months in Tanzania


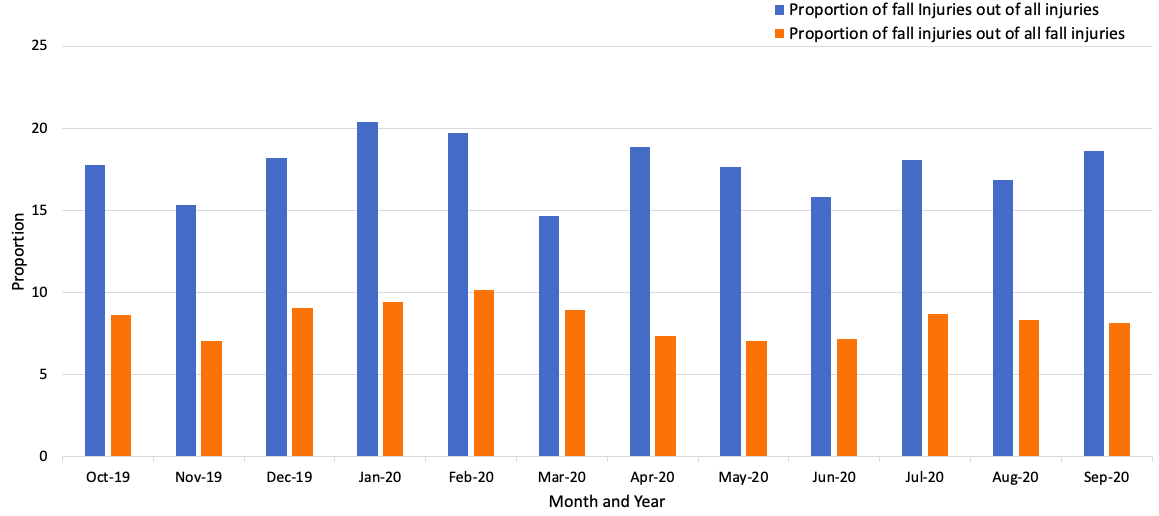

Supplement: Supplementary file 1 — Supplementary Material 1 [file 12873_2023_805_MOESM1_ESM.docx]
